# Supplementary material for: Robust Superhydrophobic Brass Mesh with Electrodeposited Hydroxyapatite Coating for Versatile Applications
Source: Molecules. 2022 Aug 31;27(17):5624. doi: 10.3390/molecules27175624 (PMC9457535; doi:10.3390/molecules27175624)
Supplement: Supplementary file 1 [file molecules-27-05624-s001.zip › molecules-1843646-Supplementary.pdf]

molecules

MDPI

Supplementary Materials

Robust Superhydrophobic Brass Mesh with Electrodeposited Hydroxyapatite Coating for Versatile Applications

Yu-Ping Zhang [1,2,*], Shi-Ming Zhang [2], Peng-Fei Liu [2], De-Liang Chen [3], Yuan Chen [3], Meng-Jun Chen [3]

1 College of Chemistry and Materials Engineering, Hunan University of Arts and Science, Changde 415000, China
2 Henan Institute of Science and Technology, Xinxiang 453000, China; zsmhnp@163.com (S.-M.Z.); lpf8856@163.com (P.-F.L.)
3 Changde Zhengyang Biotechnology Co., Ltd., Changde 415000, China; fermat3878@163.com (D.-L.C.); chenyuan626@live.cn (Y.C.); cmj_cd@163.com (M.-J.C.)
* Correspondence: beijing2008zyp@163.com

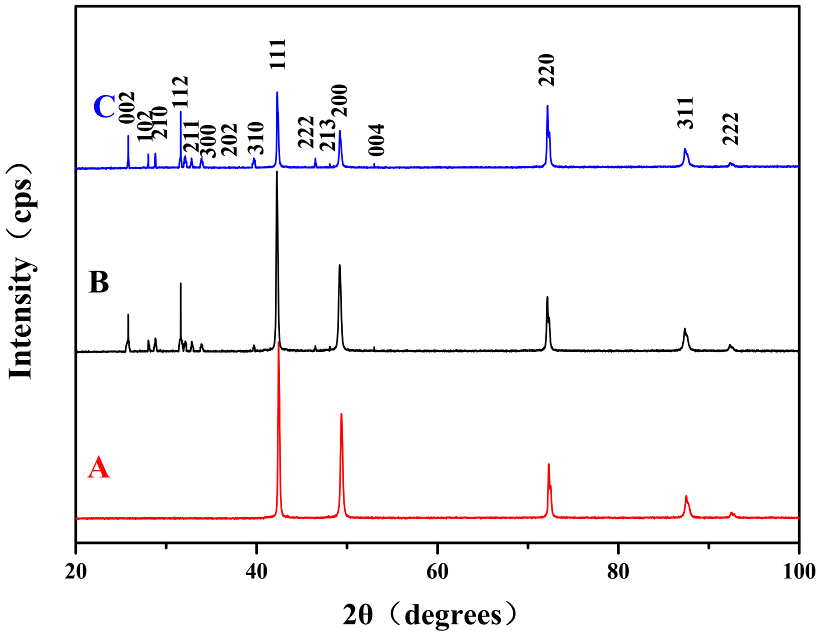

Figure S1. Characterization of the untreated, electrodeposited and superhydrophobic brass meshes by XRD. Symbols A, B and C stands for the untreated, electrodeposited and superhydrophobic brass mesh, respectively.

As shown in Figure 3A, the XRD diffractograms of the brass mesh was presented. It showed the typical characteristic diffraction peaks located at 42.2°,49.2°,72.1°, 87.3°,92.2°of 2θ, which corresponds to the crystallographic planes (111), (200), (220), (311), (222) of the standard card 00-035-1358 for brass substrate. Compared to A, the peaks at 25. 8°, 28. 0°, 28. 8°, 31. 6°, 32. 1°, 32. 8°, 33.9°, 39.7°, 46.5°, 48.1°, and 53.1° were found in Fig.3B and 3C, which correspond to the (002), (102), (210), (112), (211), (300), (202), (310), (222) (213), (004) crystal faces of the standard card (PDF09-432). The above data indicated hydroxyapatite was successfully deposited on the brass mesh.

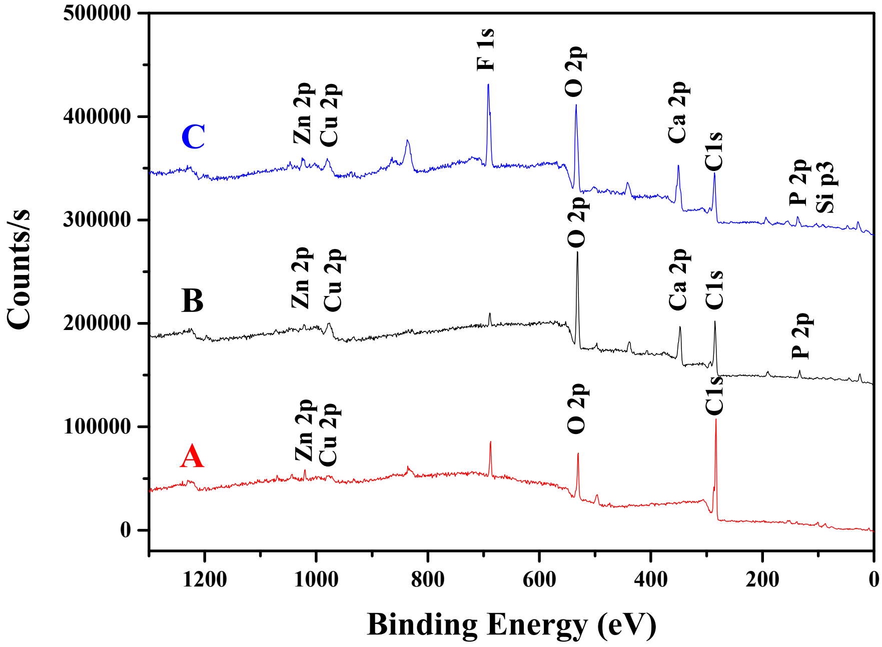

Figure S2. Characterization of the untreated, electrodeposited and superhydrophobic brass meshes by XPS. Symbols A, B and C stands for the untreated, electrodeposited and superhydrophobic brass mesh, respectively.

The chemical composition of the brass meshes were determined by XPS. In Fig.4A, the characteristic peaks Cu 2p, Zn 2p signals were detected at 976.98 eV and 1020.65 eV, which proved that the brass material is composed of copper and zinc elements. As shown in Fig.4B, the characteristic peaks Ca 2p and P 2p were found at binding energies of 365.98eV and 151.98eV, which means the material contains calcium and phosphorus elements. The characteristic peak of O 1s was found at a binding energy of 557.98 eV, and the higher intensity of the peak was observed for electrodeposited and superhydrophobic brass meshes compared with the pristine mesh. It was attributed to the forming of calcium-oxygen bond in hydroxyapatite. Two peaks of F 1s and Si 3p were found at a binding energy of 683.89 eV and 124.38 eV for the superhydrophobic brass mesh, which demonstrated a low surface energy material of FOTS was modified on the surface of electrodeposited mesh.

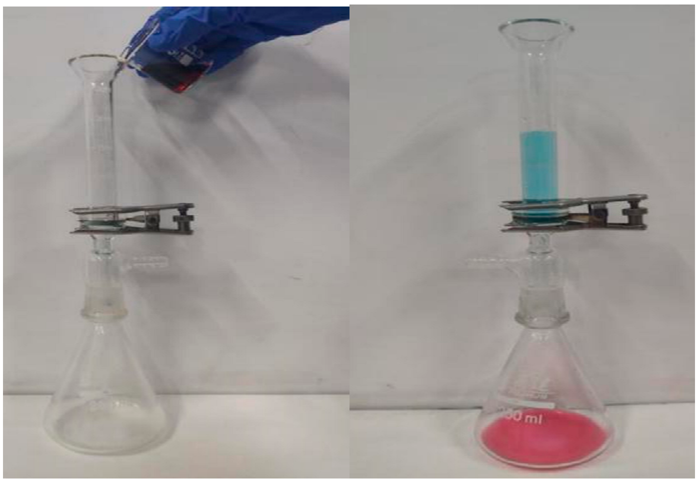

Figure S3. Oil/water separation test using the fabricated mesh.
